# Case report: Paraganglioma masquerading as angiosarcoma: diagnostic‐dilemma in vascular tumors

**DOI:** 10.3389/fonc.2024.1462956

**Published:** 2024-12-05

**Authors:** Ahilan Raj Rajoo, Saravanan Kannairan, Hisham Arshad Habeebullah Khan, Mohamad Azim Md Idris, Geok Chin Tan, Kishen Raj Chandra Sakaran, Lenny Suryani Safri

**Affiliations:** ^1^ Department of Surgery, KPJ Healthcare University, Nilai, Malaysia; ^2^ Vascular Unit, Department of Surgery, Universiti Kebangsaan Malaysia, Cheras, Malaysia; ^3^ Histopathology Unit, Department of Pathology, Universiti Kebangsaan Malaysia, Cheras, Malaysia

**Keywords:** paraganglioma, angiosarcoma, retroperitoneal tumor, CT-guided biopsy, histopathology

## Abstract

Paragangliomas originating from blood vessels are exceptionally rare, presenting diagnostic challenges due to their histological resemblance to other vascular neoplasms. We present a case study of a 60-year-old woman with underlying hypertension and dyslipidemia with obesity, initially diagnosed with angiosarcoma based on imaging and histological characteristics viewed via CT-guided biopsy. Intraoperative exploration revealed a lobulated tumor located between the inferior vena cava (IVC) and aorta measuring 7 cm × 8 cm, during which the patient developed transient hemodynamic instabilities. Histopathological examination and immunohistochemical staining using neuroendocrine markers (chromogranin, synaptophysin, S-100 protein, and CD-56) later confirmed the tumor as a retroperitoneal paraganglioma. Retroperitoneal paraganglioma was initially misdiagnosed as angiosarcoma due to the overlapping imaging characteristics between the two tumors. This highlights the importance of raising suspicion on the possibility of retroperitoneal paraganglioma when imaging examination indicates angiosarcoma and to incorporate histopathological examination and immunohistochemistry in the diagnosis to avoid misdiagnosis.

## 1 Introduction

Paragangliomas have been reported within blood vessels during vascular procedures, highlighting the unique challenges associated with their diagnosis and management (1). While paragangliomas arise from extra-adrenal chromaffin cells, angiosarcomas are aggressive malignancies that develop from vascular endothelial cells (2). Differentiating between paragangliomas and angiosarcomas is challenging due to their overlapping clinical and histological features (2). This overlap necessitates a comprehensive approach to diagnosis and management. Previous research has highlighted the difficulties in differentiating paragangliomas from other neoplasms, especially when they manifest in atypical locations like blood vessels (1). This underscores the importance of utilizing a range of diagnostic tools and techniques to accurately identify and differentiate paragangliomas from other vascular tumors. Additionally, the rarity of extra-adrenal paragangliomas contributes to a high error rate in cytological diagnoses, particularly when they present as peripancreatic masses, emphasizing the need for increased awareness among healthcare professionals regarding the diverse presentations of paragangliomas (3). Immunohistochemical studies play a crucial role in accurately diagnosing these neoplasms (2). The presence of vascular components within paragangliomas, which can resemble angiosarcomas histologically, further complicates differentiation (2). Careful examination of tumor architecture, immunohistochemical profiles, and clinical context is essential for accurate diagnosis (2). We present a case of angiosarcoma that was confirmed by histopathological examination (HPE), which was located between the aorta and inferior vena cava that turned out to be a paraganglioma.

**Figure 1:**
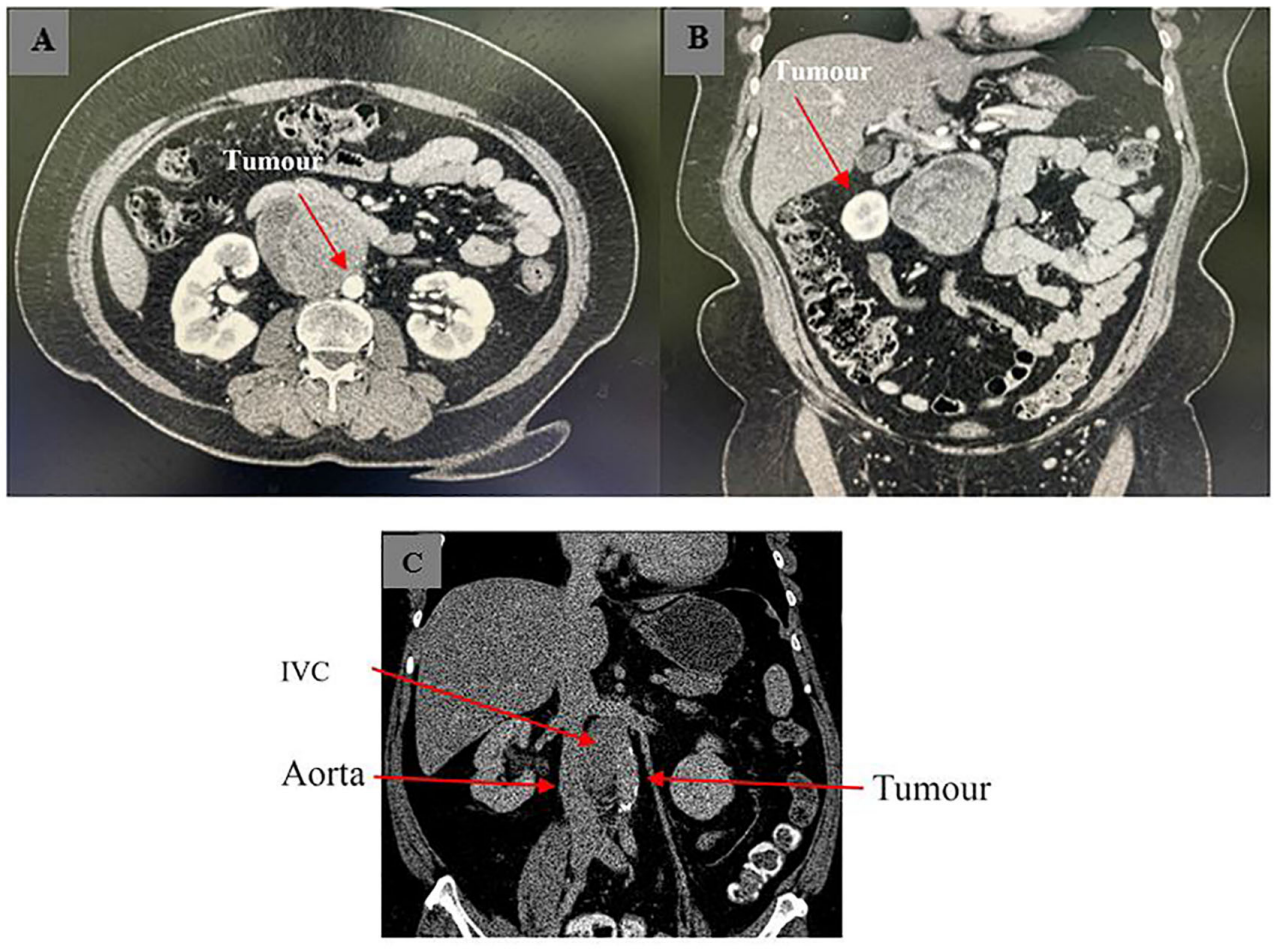
Axial **(A, B)** and coronal view **(C)** of CT imaging showing retroperitoneal tumor with close proximity to major vessels. The tumor appeared as a hypodense mass under CT imaging, suggesting necrosis within the mass.

**Figure 2:**
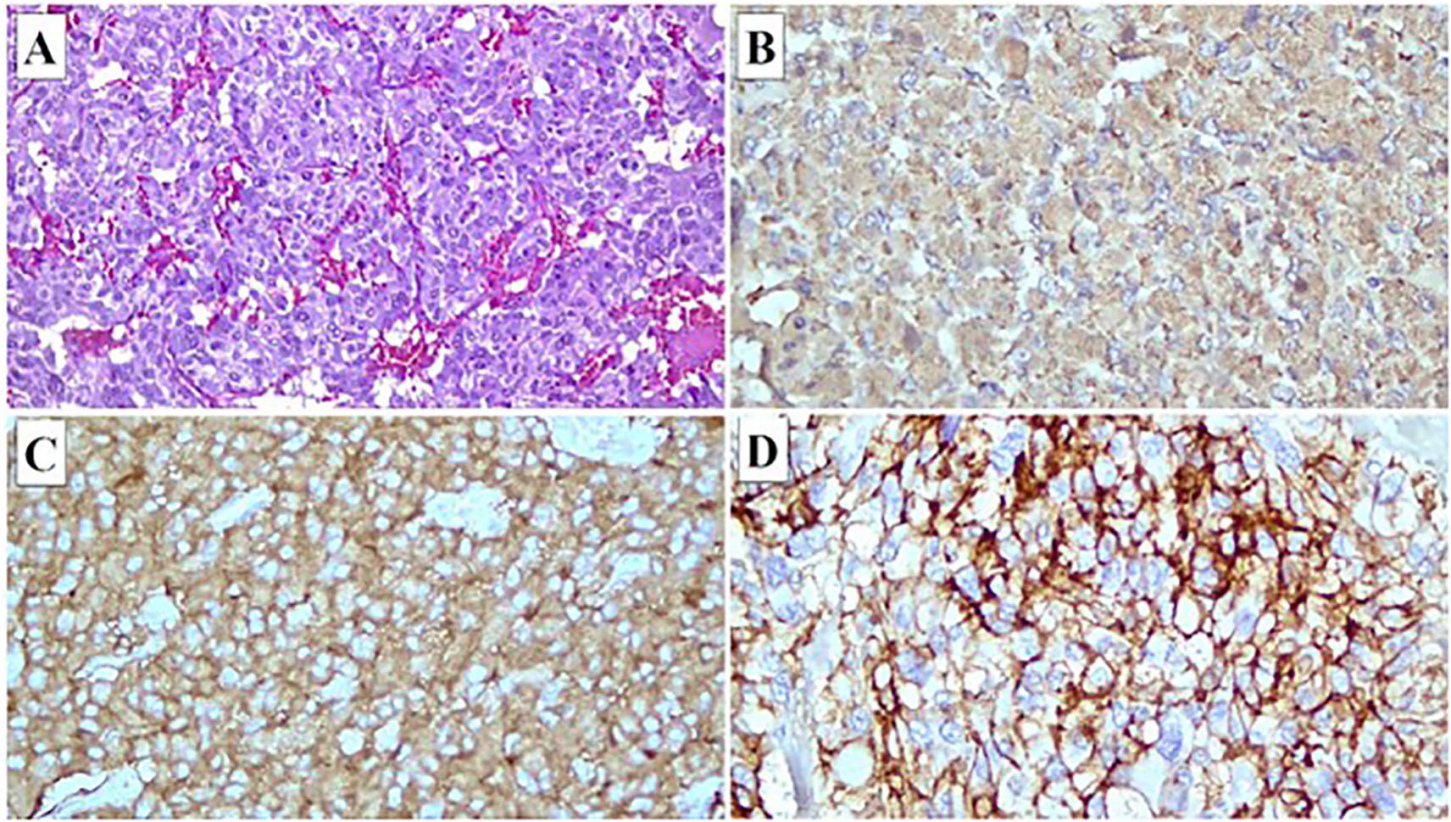
Immunohistochemical staining of tumor cells. **(A)** Hematoxylin and eosin stained section showing that the tumor is encapsulated and composed of neoplastic cells separated by thin fibrovascular septa into lobules. The neoplastic cells demonstrate pleomorphic and vesicular nuclei, open chromatin, and small prominent nucleoli. Many bizarre-shaped tumor cells are observed. Immuno-stained sections showing that the tumor cells are immunoreactive toward chromogranin **(B)**, synaptophysin **(C)**, and CD56 **(D)**.

For this case, a 60-year-old woman with underlying hypertension and dyslipidemia with obesity presented incidentally with a mass adjacent to a major blood vessel on computed tomography (CT) imaging when she went for a routine medical check-up (**Figure 1**). CT-guided biopsy suggested angiosarcoma due to its proximity to the inferior vena cava (IVC) and aorta and the appearance of necrosis inside the tumor masses, prompting surgical excision despite the patient’s asymptomatic status. Intraoperatively, a lobulated tumor located between the IVC and aorta measuring 7 cm × 8 cm was observed, during which the patient developed tachycardia and hypotension, raising suspicion of a neuroendocrine tumor. Further HPE (**Figure 2**) revealed the tumor to be a retroperitoneal paraganglioma because not only did the tumor cells appeared as spindle-shaped cells clustered with the sustentacular cells into small nests, but they were also immunoreactive toward chromogranin, synaptophysin, and CD56. Postoperative recovery was uneventful.

## 2 Discussion

Paraganglioma is a rare tumor that arises from extra-adrenal chromaffin cells (4). Its prevalence ranges from ½,000 to 1/6,500 (4). Paraganglioma originates from paraganglia located at several anatomical sites, including the head, neck, thorax, and abdomen. It is characterized by secretion of excessive catecholamines such as epinephrine, norepinephrine, and dopamine, which causes clinical symptoms like episodic hypertension, tachycardia, and diaphoresis (4). Among all types of paragangliomas, retroperitoneal paraganglioma accounts for a prevalence between 21.5% and 87% (5).

In the case of the presented patient, initial diagnosis based on CT-guided biopsy indicated angiosarcoma. This was due to the imaging and histological characteristics observed that were highly similar to those that might have appeared in the case of angiosarcoma: soft-tissue masses in the sympathetic chains associated with the aorta, cystic degeneration, and sizeable areas of necrosis and hemorrhage inside the masses and a marked peripheral enhancement in the arterial phase (6). The proximity of the hypodense mass to the aorta and IVC viewed under CT imaging led physicians to initially diagnose the necrotic-looking tumor as angiosarcoma.

However, the diagnosis of angiosarcoma was later ruled out when further HPE revealed a characteristic of neuroendocrine tumor: tumor cells appeared as polygonal or spindle-shaped cells clustered with the sustentacular cells into small nests or alveoli characteristically known as “Zellballen,” surrounded by a rich vascular network; cytoplasm of the cell had a finely granular appearance, and the nuclei appeared round or ovoid with a stippled “salt and pepper” chromatin (7).

Diagnosis of retroperitoneal paraganglioma was further confirmed through immunohistochemical staining that showed positivity for chromogranin, synaptophysin, S-100 protein, and CD-56, and negativity for smooth muscle actin (SMA) (5, 7). Angiosarcoma was ruled out mainly based on the diffuse positivity for the neuroendocrine markers (chromogranin, synaptophysin, S-100 protein, and CD56) together with a negative cytokeratin reaction (SMA) and, most importantly, a positivity for CD56 (7), thus arriving at the final diagnosis of retroperitoneal paraganglioma.

Misdiagnosis of retroperitoneal paraganglioma should be avoided prior to surgery, as intraoperative manipulation of the tumor may cause a secretion of catecholamines, which may give rise to challenges in maintaining hemodynamics (7). Since retroperitoneal paraganglioma is rare, its diagnosis remains complex, and many misdiagnosed cases of the tumor have previously been reported (5, 8, 9). The current case presents an example of retroperitoneal paraganglioma being misdiagnosed as angiosarcoma due to the similarities in CT imaging and histological features between the two tumors. In addition, the case was also being misdiagnosed due to the negligence of making diagnostic decision simply based on HPE alone instead of incorporating other laboratory tests. Therefore, it is recommended to incorporate immunohistochemistry tests in the diagnosis of tumors, especially in the case of angiosarcoma, as CT imaging and HPE can be rather misleading due to the overlapping characteristics between different types of tumors.

## Data Availability

The original contributions presented in the study are included in the article/supplementary material, further inquiries can be directed to the corresponding author/s.
